# Relation between the size of patent foramen ovale and the volume of acute cerebral ischemic lesion in young patients with cryptogenic ischemic stroke

**DOI:** 10.1007/s10072-021-05330-y

**Published:** 2021-05-29

**Authors:** Federica Benvenuti, Francesco Meucci, Luisa Vuolo, Rita Nistri, Giovanni Pracucci, Antonella Picchioni, Gabriele Venturini, Miroslava Stolcova, Ylenia Failli, Patrizia Nencini, Carlo Di Mario, Cristina Sarti, on behalf of Heart and Brain Team Careggi University Hospital, Florence, Italy

**Affiliations:** 1 NEUROFARBA Department, University of Florence, Florence, Italy; 2 Structural Interventional Cardiology, Careggi University Hospital, Florence, Italy; 3 Neuroradiology Unit, Careggi University Hospital, Florence, Italy; 4 Department of Heart and Vessels, Careggi University Hospital, Florence, Italy; 5 Stroke Unit, Careggi University Hospital, Florence, Italy

**Keywords:** Patent foramen ovale, Cryptogenic stroke, Cerebral lesion volume, Echocardiographic patent foramen ovale dimensions, Cerebral magnetic resonance

## Abstract

**Background:**

Patent foramen ovale (PFO) closure is superior to medical therapy alone to prevent stroke recurrence in selected patients. Small cortical infarcts and large right to left shunts seem to identify patients who will benefit most from closure. We aimed to study the correlation between the size of the PFO and the volume of cerebral ischemic lesions in young patients with cryptogenic ischemic stroke.

**Methods:**

PFO dimensions and acute ischemic lesion volume of 20 patients, aged<55 years, were analyzed with transesophageal echocardiography and brain magnetic resonance imaging, respectively. The association between the volume of ischemic lesions with the length of PFO, maximum separation between septum primum and septum secundum, and the combination of the twos was explored.

**Results:**

A direct statistically significant correlation was found between cerebral lesion volume and maximum separation of septum primum and septum secundum (p=0.047). Length of PFO showed a non-significant trend towards an inverse correlation with lesion volume (p=0.603). Multiple linear regression analysis showed that cerebral lesion volume was dependent directly on maximum separation and inversely on length of PFO (regression coeff. −0,837; p= 0.057; 2,536, p=0.006, respectively).

**Conclusions:**

These data suggest that even small PFO might be pathogenetic in case of small cerebral infarcts and that large cerebral infarcts might be PFO related if the shunt is large. If confirmed, the combination of detailed characteristics of PFO with the volume of cerebral infarct could be integrated in a new score to select patients who would take real advantage from a percutaneous closure.

## Background

Patent foramen ovale (PFO) is associated with cerebral ischemic stroke in case-control studies [1] and recent randomized trials show that its closure is more effective than medical therapy alone to reduce stroke recurrence in selected patients [2].

Consensus statements [3] indicate that the morphology of PFO and the characteristics of the cerebral ischemic lesions are key factors to reach this goal, specifically large shunts associated with atrial septal aneurism and small cortical cerebral infarcts [4]. It is not clear how to manage small PFOs and large ischemic lesions.

In a context of scarce and conflicting evidence [5, 6], we aim to investigate a possible relationship between PFO size and acute cerebral ischemic lesion volume in young patients with cryptogenic stroke.

## Methods

### Patient population

From January 2000 to August 2018, we enrolled consecutive patients, aged <55 years, with PFO, admitted to the Stroke Unit of Careggi University Hospital (Florence) for acute ischemic stroke of undetermined aetiology. Patients were classified as cryptogenic stroke according to TOAST classification [7]; the diagnostic work-up used was the one proposed by Saver [8]. We excluded 12 patients who underwent acute phase recanalization treatments that could influence cerebral lesion volume and 38 patients for whom transesophageal echocardiography (TEE) recorded images or magnetic resonance (MR) images were not available. The clinical description of these latter patients is shown in Table 1.

**Table 1:** Clinical characteristics of excluded patients

	N=38
Sex (Female) %	47.4
Mean age, (range)	39.5±8.1 (18.5–49.9)
Hypertension (%)	13.1
Diabetes (%)	2.6
Dyslipidemia (%)	28.9
Smoking (%)	34.2
Obesity (%)	5.3
Estroprogestinics (%)	44.4
Migraine (%)	21
Previous stroke (%)	5.3
NIHSS mean (range)	3.61 (1–23)

**Fig. 1:**
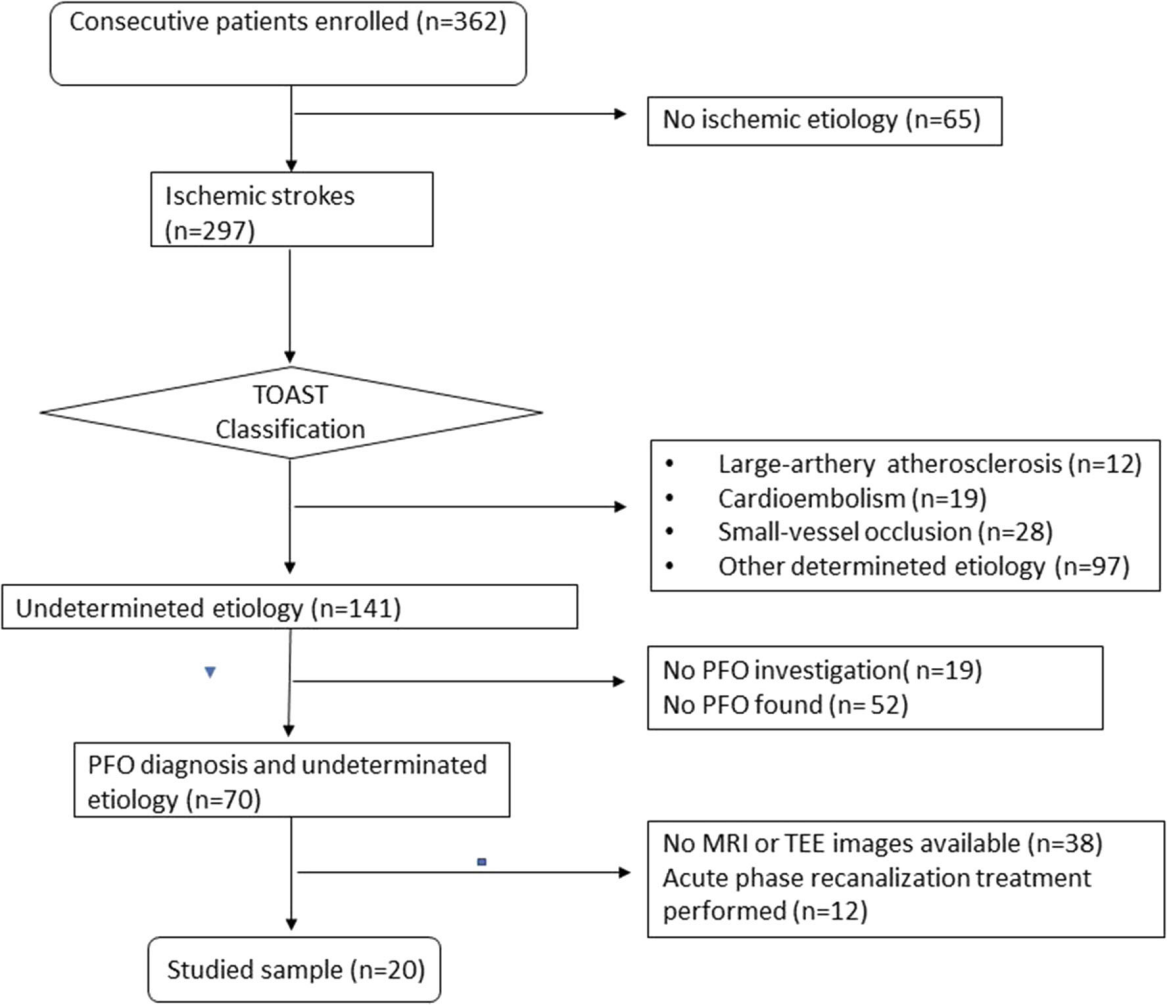
Flowchart of patients’ enrolment (January 2000–August 2018)

Twenty patients satisfied these criteria (Fig. 1).

Ethical approval was waived by the local Ethics Committee of University of Florence in view of the retrospective nature of the study, and all the procedures being performed were part of the routine care. The study was undertaken with the understanding and written consent of each subject and conforms with World Medical Association Declaration of Helsinki.

### MRI

**Fig. 2:**
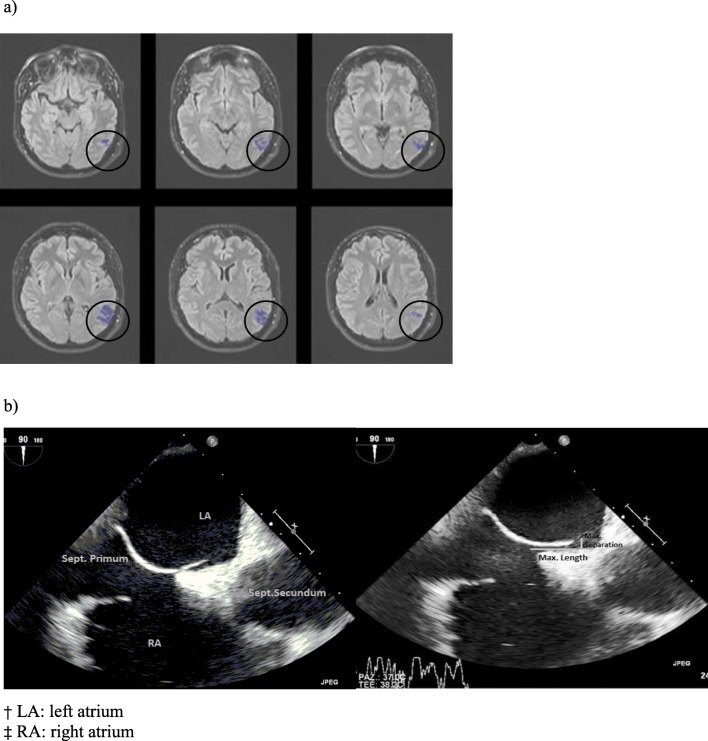
**a** Magnetic resonance images: ischemic lesion volume, FLAIR sequences; **b** TEE images: patent foramen ovale dimensions. † LA: left atrium, ‡ RA: right atrium

MR images were analyzed by two collaborating operators (L.V. neuroradiologist, F.B. medical student). Acute strokes were confirmed through the analysis of the diffusion-weighted images (DWI) and fluid-attenuated inversion recovery (FLAIR) sequences. Volume of the cerebral ischemic lesion was measured using multi-vendor Olea Sphere 3.0 software (Fig. 2a).

### TEE

PFO were diagnosed by TEE. Contrast study with agitated saline bubble solution was conducted to estimate the degree of right-to-left shunt, and the number of bubbles seen in the left atrium after 3 cardiac cycles was recorded [9]. We classified shunt as mild (<10 bubbles), moderate (≥ 10 bubbles), and severe (when bubbles appear uncountable due to the high amount) [9]. Presence of atrial septal aneurism (ASA) was also recorded.

We measured the PFO dimensions through Horos software using aortic short axis and bicaval echocardiography projections. We specifically considered (1) the maximum length, in terms of the greatest overlapping of septum primum and septum secundum (mm) and (2) the maximum separation between septum primum and septum secundum (mm) (Fig.2b).

### Statistical analysis

Data were analyzed using the statistical programme SPSS, through a descriptive analysis of the characteristics of the sample with cryptogenic stroke and PFO, with percentage frequency for the categorical variables and mean and standard deviation for numerical variables. Pearson correlation was used to evaluate the association between single dimensions (length and maximum separation) of PFO and volume of ischemic lesions, and a linear regression analysis was performed to evaluate the predictive value of the association between the two dimensions of the PFO on cerebral ischemic lesion volume.

## Results

Sample characteristics are shown in Table 2. Mean NIHSS on admission was 3.6 (range 1–24); 75% of patients were independent at follow-up. Smoking and dyslipidaemia were the most represented vascular risk factors. Elevated frequency of estroprogestinic use was present in females (44.4%).

**Table 2:** Sample characteristics

	N=20	%
Sex (female)	9	45
Mean age (range)	38,8 years	(19–49)
NIHSS, mean (range)	3.6	(1–24)
3 months mRS n (%)		
0–1	15	75
2	2	10
3	3	15
Hypertension n (%)	2	10
Diabetes n (%)	2	10
Dyslipidemia n (%)	6	30
Smoking n (%)	7	35
Obesity n (%)	4	20
Estroprogestinics n (%)	4/9	44.4
Migraine n (%)	3	15
Previous stroke n (%)	1/19	5.3

Echocardiographic and cerebral MRI findings are shown in Table 3. Significant shunt was present in 55% of patients, mean length of PFO was 9.85 mm with a range between 1.03 and 18 mm, and mean maximum separation was 3.58 (range 1.03–9.53). Cerebral lesion volume showed a high variation ranging from very small (0.04 cm^3^) to very large lesions (26.59 cm^3^) with a mean of 4.07 cm^3^.

**Table 3:** Echocardiographic and MRI findings

Echocardiographic findings	Mean	Range
PFO length (mm)	9.85	1.03–18
PFO maximum separation (mm)	3.58	1.03–9.53
PFO Shunt entity	N	Frequency
•<10 microbubbles	2	10%
•≥10 microbubbles	7	35%
•Uncountable	11	55%
ASA entity	N	Frequency
•10–15mm	3	15%
•>15mm	3	15%
•Not found	12	60%
•Unknown	2	10%
MRI findings	Mean	Range
Ischemic lesion volume (cm^3^)	4.07	0.04–26.59

**Fig. 3:**
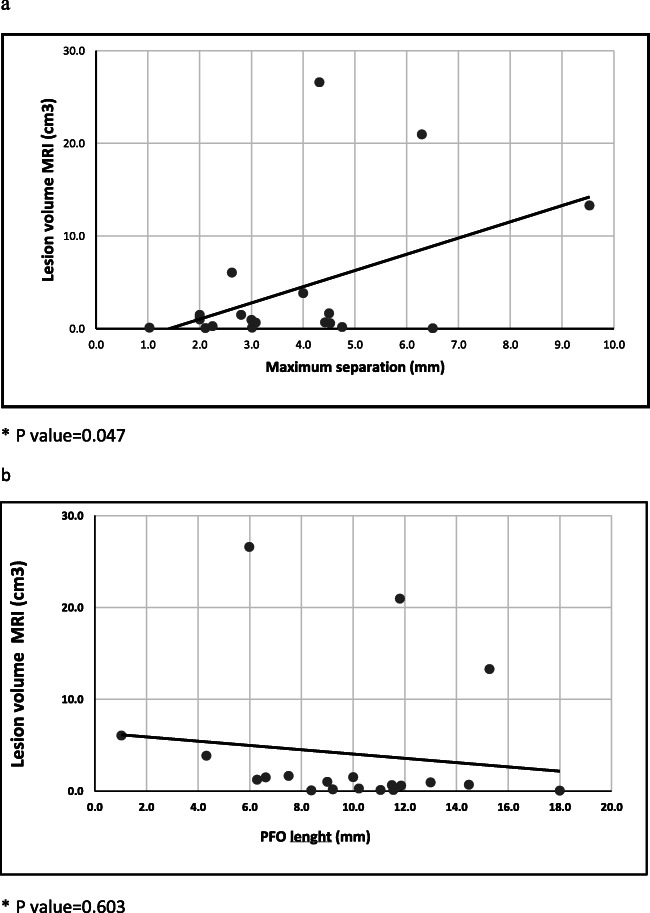
Correlation between cerebral ischemic lesion volume and: **a** Maximum separation of septum primum and septum secundum; **b **PFO length

We found a statistically significant correlation between cerebral lesion volume and maximum separation between septum primum and septum secundum (p=0.047) (Fig. 3a). Length of PFO shows a non-significant trend towards an inverse correlation with lesion volume (p=0.603) (Fig. 3b). The linear regression analysis, carried out using the dimensions of PFO as predictive variable and volume of cerebral lesion as dependent variable, showed that cerebral lesion volume is directly dependent on maximum separation and inversely dependent on length of PFO (linear regression: length of PFO regression coeff. −0,837; p= 0.057; maximum separation regression coeff. 2,536, p=0.006).

## Discussion and conclusion

In clinical practice, we face different kinds of patients regarding embolic ischemic stroke and PFO that can be grouped as follows:

1. Patients with probable PFO-related stroke
2. Patients with possible PFO-related stroke
3. Patients with cryptogenic stroke and PFO

Assigning a patient to one of these categories is not an easy task. In the first category, we would include patients with embolic ischemic stroke, presence of or clear factors predisposing to a deep vein thrombosis, and large PFO associated or not with atrial septal aneurysm. In the second category, we would include patients with cryptogenic embolic ischemic stroke, large PFO associated or not with atrial septal aneurysm, absence of deep vein thrombosis, and predisposing factors.

In the third category, we would include all those patients with cryptogenic embolic ischemic stroke and small PFO without atrial septal aneurism, without deep vein thrombosis nor predisposing factors. Whether or not to propose PFO closure to such patients is unclear, particularly after the results of the clinical trials suggesting the greatest benefit in the presence of a large PFO.

Another group of patients worthy to be accurately discussed is represented by those with large infarcts considering that literature data show that the typical cerebral ischemic lesion correlated to PFO is mainly represented by small cortical one [4].

In the presence of small PFO and in case of large cerebral ischemic lesions, the search of a pathogenic mechanism other than PFO must be even more accurate than ever, but if at the end of an appropriate/standardized screening of the patient [8] we conclude for a true cryptogenic stroke with PFO, we need some elements to decide whether to propose PFO closure or not.

Our study aimed to preliminary explore the existence of a relationship between PFO size and cerebral ischemic lesion volume. In literature, we found two studies with the same aim but with conflicting results: Jung et al. [5] observed a positive correlation between PFO size and cerebral infarct lesion, while Akhondi et al. [6] found no significant association between PFO dimensions and brain infarct volume.

Our results give some hints worthy to be further explored.

The direct correlation between cerebral lesion volume and the dimensions of PFO in terms of maximum separation between septum primum and septum secundum points out the importance of not excluding the pathogenic role of those PFOs characterized by small dimensions when associated with limited cerebral lesions. In the meantime, large ischemic lesions can be PFO-related when associated with a large PFO. Otherwise, the association between large ischemic lesion volume and small PFOs may guide the clinician to delve into other possible stroke causes. The trend towards an inverse correlation between PFO length and volume of cerebral lesion can suggest a ‘filter-like’ action of long tunnel on larger emboli, closely related to the length and morphology of the tunnel itself.

The linear regression analysis carried out using the dimensions of PFO as predictive variable and volume of cerebral lesions as dependent variable showed that cerebral lesion volume is directly dependent on maximum separation and inversely dependent on the length of PFO; that is, shorter and larger PFO seem to provide a higher embolic risk. Such PFOs may be somehow hemodynamically assimilated to an interatrial defect.

We should underline that our study may be considered a pilot study, because of the small sample we managed to analyze. Moreover, PFO dimensions were measured by transoesophageal echocardiography that is an operator-dependent procedure. To allow us to have a better estimation of the parameters, it could be useful in subsequent studies to use intraoperative sizing balloons in those patients who undergo percutaneous procedure.

If these data are confirmed in studies with a larger sample of patients, they could become part of the flourishing research scenario of the pathogenetic role of PFO in ischemic stroke, integrating the existing pathogenic score [10, 11].
